# Microbial Resistance Movements: An Overview of Global Public Health Threats Posed by Antimicrobial Resistance, and How Best to Counter

**DOI:** 10.3389/fpubh.2020.535668

**Published:** 2020-11-04

**Authors:** Sameer Dhingra, Nor Azlina A. Rahman, Ed Peile, Motiur Rahman, Massimo Sartelli, Mohamed Azmi Hassali, Tariqul Islam, Salequl Islam, Mainul Haque

**Affiliations:** ^1^School of Pharmacy, Faculty of Medical Sciences, The University of the West Indies, St. Augustine, Trinidad and Tobago; ^2^Department of Physical Rehabilitation Sciences, Kulliyyah of Allied Health Sciences, International Islamic University Malaysia, Kuantan, Malaysia; ^3^Warwick Medical School, University of Warwick, Coventry, United Kingdom; ^4^Oxford University Clinical Research Unit, Wellcome Trust Asia Programme, The Hospital for Tropical Diseases, Ho Chi Minh City, Vietnam; ^5^Centre for Tropical Medicine and Global Health, Nuffield Department of Medicine, Oxford University, Oxford, United Kingdom; ^6^Department of General and Emergency Surgery, Macerata Hospital, Macerata, Italy; ^7^The Discipline of Social and Administrative Pharmacy, School of Pharmaceutical Sciences, Universiti Sains Malaysia, Minden, Malaysia; ^8^UChicago Research Bangladesh, Dhaka, Bangladesh; ^9^Department of Microbiology, Jahangirnagar University, Dhaka, Bangladesh; ^10^The Unit of Pharmacology, Faculty of Medicine and Defence Health, National Defence University of Malaysia, Kuala Lumpur, Malaysia

**Keywords:** antibiotics, antimicrobial resistance, AMR, globe, public health, hazard

## Abstract

Antibiotics changed medical practice by significantly decreasing the morbidity and mortality associated with bacterial infection. However, infectious diseases remain the leading cause of death in the world. There is global concern about the rise in antimicrobial resistance (AMR), which affects both developed and developing countries. AMR is a public health challenge with extensive health, economic, and societal implications. This paper sets AMR in context, starting with the history of antibiotics, including the discovery of penicillin and the golden era of antibiotics, before exploring the problems and challenges we now face due to AMR. Among the factors discussed is the low level of development of new antimicrobials and the irrational prescribing of antibiotics in developed and developing countries. A fundamental problem is the knowledge, attitude, and practice (KAP) regarding antibiotics among medical practitioners, and we explore this aspect in some depth, including a discussion on the KAP among medical students. We conclude with suggestions on how to address this public health threat, including recommendations on training medical students about antibiotics, and strategies to overcome the problems of irrational antibiotic prescribing and AMR.

## Introduction

In the pre-antibiotic era, contagious microbial diseases such as smallpox, cholera, diphtheria, pneumonia, typhoid fever, plague, tuberculosis, typhus, syphilis, etc. were widespread all over the globe (1), and infectious diseases were the primary cause of morbidity and mortality (2). Until relatively recently, the average life span was limited to 4 or 5 decades, in part because minor infectious diseases often progressed to septicemia and death (3–5). The discovery of penicillin by Sir Alexander Fleming in 1928, completely transformed medical practice., It still ranks as the most effective life-saving intervention in medicine, saving millions of lives in the years to follow (6, 7). The discovery of antibiotics reduced the death rate by 25–30% for both community-acquired and healthcare-associated pneumonia, 75% for endocarditis, 60% for meningeal or cerebral infections, and 11% for cellulitis (8, 9).

Unfortunately, within 50–60 years, these successes of medical science were starting to fade as micro-organisms began to develop resistance toward antibiotics (10–12). Thereafter, the fight against pathogens has been a losing battle despite relentless attempts by scientists to develop new and more effective agents against the micro-organisms. Although antimicrobial resistance (AMR) is a natural phenomenon, the process has been much enhanced because of the overuse, underuse, and misuse of antimicrobials both in humans and animals (10–13). Consistent expansion of AMR has stunted both the prevention and treatment of infections caused by bacteria, parasites, viruses, and fungi (14). As a result, AMR creates a progressively grave risk for global public health in the last two decades and has now been appraised as the highest health endangerment in the 21st centenary (14, 15). In the United States of America (USA), the healthcare cost related to AMR was $55 billion per year in 2013, and every year more than 2 million of the US population are infected with resistant infections, which claim over 23,000 deaths (16). The European Center for Disease Prevention and Control (ECDC) and European Medicines Agency (EMEA) jointly estimated that in 2009, the overall annual healthcare expenditure and productivity losses were over €1.5 billion because of the additional cost to AMR infectious disease management. Additionally, hospital costs were estimated to be over €900 million, and multidrug-resistant (MDR) microbial infections claimed around 33,000 lives (17–19). MDR was demarcated as resistance to at least one agent in three or more antimicrobial classes, extensively-drug-resistant (XDR) was delineated as non-sensitivity to at least one agent in all but two or fewer antimicrobial groups (i.e., bacterial isolates remain susceptible to only one or two categories) and pan-drug-resistant (PDR) was described as resistance to all medicines in all antimicrobial classifications (20). Furthermore, multiple studies reported that patients and parents of pediatric patients were ill-informed about AMR and lacked understanding of how resistance operates for treatment difficulty and failure. This lack of understanding leads to a failure of the public to recognize any responsibility for tackling the issue of AMR (21, 22).

## Aims and Methods

This narrative review draws on the relevant literature around the history of antimicrobial development in the context of emerging drug resistance and antimicrobial prescribing worldwide, in order to characterize the global public health threat to the prevention and control of infectious diseases with their associated morbidity, mortality, hospitalization, increased hospital stay, and healthcare cost.

We hope that by raising awareness of the issues and by discussing educational approaches to reducing antimicrobial resistance we can contribute to reducing patient miseries and improving patient care.

The review is based on published literature available to the Universiti Pertahanan Nasional Malaysia (UPNM, National Defense University of Malaysia), searches were carried out using SCOPUS, EBSCO, PubMed, and Google Scholar. The study was conducted between September-2-2019 to June-23-2020. Search terms were antibiotics, history, discovery, penicillin, golden era, challenges, obstructing, resistance, development, 1960–2000, combinations agents, 2015–2020, antimicrobial resistance, AMR, dangerous, pathogenic microbes, poor development, mechanisms, microbial resistance, rational, use, irrational, prescribing, developed, developing, countries, medical doctors, medical students, undergraduate medical course, curriculum, knowledge, attitude, practice, strategies, combat, combat antimicrobial resistance, globe, public health, risk. Relatable and appropriate journals were pinpointed through hand-searched, and references were checked to find other related published manuscripts.

## History of Antibiotics in the Last Millennium

The word “antibiotics” was first coined in 1941 to describe the antibacterial drugs by Professor Selman Waksman, who discovered over 20 antibiotics (23, 24), paving the way for antimicrobial drugs to becoming one of the most efficacious forms of pharmaceutical agents in the history of modern medicine (25–27). Even earlier, Paul Vuillemin used the word “antibiose” in 1890 to describe an agent that antagonizes the activity of diverse microorganisms (28–30). Despite the widespread belief that antibiotics have only been utilized in the past century, recent evidence suggests that substances with antimicrobial activity were consumed a few thousand years back (31–34). Prehistoric people used a diversity of natural substances such as herbs, honey, and even animal feces as the remedy for infection (35–37). Fungal infected bread was successfully used in treating skin infections around the globe in ancient times, particularly in Egypt, China, Serbia, Greece, and Rome (37). The concept of using moldy bread was mentioned in 1640 when John Parkinson (1567–1650) applied “fungated” bread to his patient's skin (37). The first antibiotic in the form of pure hard-sparkling crystal was mycophenolic acid, isolated by an Italian physician and microbiologist, Dr. Bartolomeo Gosio, in 1893 from *Penicillium glaucum* (later known as *P. brevicompactum*) (38, 39). Mycophenolic acid was initially thought of as a bacteriostatic agent toward *Bacillus anthracis* and additionally showed antiviral, antifungal, antitumor, and anti-psoriasis properties (38–40). This basic and essential finding was disregarded until mycophenolic acid was found again in 1913 in the USA. The lack of attention can probably be attributed to language limitation, as the original findings were published in Italian (41). Another claim to be the first antimicrobial utilized in human infection is made for pyocyanase, which was developed by German scientists in the late 1890s from green bacterial isolate (42–44).

### Discovery of Penicillin

The breakthroughs in microscope design and their mass production in the 19th Century were vital to the study of microbe activity by bacteriologists, and crucial to the discovery of Penicillin in 1928 by Alexander Fleming, a Scottish bacteriologist working at St. Mary's Hospital in London. Upon his return from a 2-week vacation, he started cleaning his table when he noticed a petri dish comprising of staphylococcus culture left on a laboratory worktable, not in the incubator as planned. He found a fungal growth in the petri dish of *Staphylococcus* spp. culture, probably accidentally contaminated as a window of the laboratory had remained open (45). Professor Fleming detected that the staphylococcus growth near the fungal clusters was becoming extinct, as demonstrated by the liquefying and clearing of the neighboring agar gel (45, 46). He started experimenting with the fungal growth and identified it as the *Penicillium* genus, which liberates a compound that could inhibit several gram-positive pathogens responsible for scarlet fever, pneumonia, gonorrhea, meningitis, and diphtheria (45). Fleming published these findings in the British Journal of Experimental Pathology in 1929 (46). The first recorded administration in humans was a single case in 1941, at which time there was too little available to give the infected policeman a full course and he died (47). Ernst Chain and Howard Florey then isolated the pure form of penicillin in the form of penicillin G, and production was scaled up, making the antibiotic available for increasingly widespread clinical use from 1942, initially for allied force military community, but was extensively marketed for civilians after the Second World War (1, 48). Alas, the first evidence of penicillin resistance was also reported around this time. In 1945, Sir Alexander Fleming, with Sir Howard Florey and Ernst Chain, were awarded the Nobel Prize in Physiology/Medicine for the invention of penicillin to treat several infectious diseases (45, 48). Nevertheless, Fleming wrote, “I did not invent penicillin. Nature did that. I only discovered it by accident” (45).

### Golden Era of Antibiotics

The introduction of penicillin and streptomycin in 1928 and 1943, respectively, presaged the time when antibiotics became a dominant component of medical care for infectious diseases. By 1950–1960 several more antimicrobials had been discovered (49). Indeed, 1930–1962 was often considered as the golden age of antimicrobial inventions as 20 different novel classes of antimicrobials were invented and developed rapidly within these two decades. Many of these went on to serve mankind for another 60 years (49–53).

A valuable introduction was the first aminoglycoside, streptomycin. This highly effective, broad-spectrum antimicrobials with a mechanism of action through inhibition of microbial protein synthesis was developed by the US scientists Waksman, Schatz, and Bugie (54, 55) from *Streptomyces griseus* and clinically first used in 1944 (56).

### Development of Antimicrobials 1960-2000

In the last 40 years of the 20 century a lot of new antimicrobials molecules came to market and collectively they contributed a lot to saving human life. Four successive generations of quinolones were developed with identical pharmacodynamics, but with incremental advantages in pharmacokinetics (57, 58).

Aminoglycoside development also saw significant advances. From streptomycin (1943), neomycin (1949), kanamycin (1957), came gentamicin in 1963. This is still in widespread use around the globe. Netilmicin followed (1967), and tobramycin (1974), and amikacin (1976) (56). All these aminoglycosides acted like streptomycin, suppressing microbial protein synthesis by way of irretrievable binding with the 30S microorganism ribosome (59).

Teicoplanin, a glycopeptide antimicrobial, which was first identified in the mid-1970s, acts by inhibiting cell wall synthesis, almost like vancomycin. Pharmacodynamically, teicoplanin does not belong to a novel class as these drugs are still glycopeptide, membrane biosynthesis inhibitors (60–63). Use of teicoplanin is likely be affected by the local prevalence of MRSA (64–66).

Although cephalosporins were first invented in 1945, they were not clinically utilized until 1964 (67). There are five generations of cephalosporins currently available in medicine (68, 69), all belonging to a class of β-lactam antimicrobials acting (like penicillin) by cell wall synthesis inhibition (69, 70). The reason why successive cephalosporin molecules appeared on the medicine-market was their incremental pharmacokinetic advantages. Cefepime—a fourth-generation cephalosporin first appeared for clinical practice in 1994, for the management of moderate-to-severe infectious disease conditions, specifically for pneumonia, straightforward and difficult urinary tract infections (UTIs), skin and soft-tissue infections, intra-abdominal infections, and febrile neutropenia (71). Cefepime resistance has since been widely reported (72–75).

The carbapenems (doripenem, ertapenem, imipenem, and meropenem) are architecturally correlated to other β-lactam antimicrobials (63). These novel antimicrobials belong to -Lactam & other cell walls- & membrane-active group, thereby, mechanism of actions remain like other cell-wall inhibitors (63). Imipenem was the first agent clinically used in the carbapenem family that was developed in the United States in 1985. Carbapenems hold the broadest spectrum of activity, and proved to be extremely potent against Gram-positive and Gram-negative microbes. The carbapenems often appear in formularies as the named and considered as the “final rescuer” or antimicrobial of last resort, reserved as a potential lifesaver for patients with life-threatening infection by highly resistant pathogens (76–80). The first reports of imipenem and meropenem resistance to Klebsiella pneumoniae appeared in 2001 (81). Sadly, recent years have seen wider emergence of MDR pathogenic microorganisms that utterly evade even this last resort of infectious disease management (82).

Amoxicillin is a penicillin which has been a widespread market success over the years, alone or in combination with clavulanic acid. However, having the same mechanism of action as other penicillins, widespread drug resistance has now limited its use (63). Clavulanic acid, like tazobactam and sulbactam, is a pharmaceutical agent utilized with antimicrobials. Nevertheless, these agents are not considered as antimicrobials, rather as microbial β-lactamases enzyme inhibitors (83).

Erythromycin was the first of a group of the macrolide group of antibiotics and was first used in 1952. Later drugs in this group, usually demonstrating some pharmacokinetic advantage, (84, 85) included clarithromycin, azithromycin, and roxithromycin. Macrolides' basic mechanism of action is similar to the earlier antimicrobials working to inhibit microbial protein synthesis by binding to bacterial 50S ribosomal subunit (86).

Trimethoprim, first used in 1962, is primarily used in urinary tract infections (87). The mode of action is to bind to dihydrofolate reductase and thereby inhibit the reduction of dihydrofolic acid (DHF) to tetrahydrofolic acid (THF), an essential precursor in the thymidine synthesis pathway. This interference inhibits bacterial DNA synthesis (88). In the early days, trimethoprim was commonly used in combination with sulfamethoxazole which inhibits dihydropteroate synthase, an enzyme involved further upstream in the same pathway. The hope was that the two agents would, by their possible synergistic effects, reduce the development of resistance (88–90). However, resistance to the combination became significant, and the serious potential adverse effects of the sulfonamide resulted in the single agent proving to be more popular in the long-term. Drug resistance is still a big problem for trimethoprim (91).

The quinolones are a good example of drugs invented and gradually refined over this period and still in widespread use across the world despite encountering antimicrobial resistance. Useful advances also comprised new formulations and many of that generation of injectables are also still in use (92–94).

Thus, these four decades, although responsible for significant incremental improvement of antimicrobial drugs in existing classes, saw little by way of fundamental innovation. It is hardly surprising that when microbes are attacked in similar ways to those which have already been circumvented, they rapidly manage to develop resistance to similar “me-too” agents. However, since 1962, only two new classes of antimicrobials have been developed (50, 95–97). Of these new classes of antimicrobials, only three were marketed for human infectious diseases, and one class was restricted just for topical administration (98).

Historical antimicrobials development is depicted in Table 1, which lists the year in which the first drug of a class was developed.

**Table 1:** Novel classes of antibiotics developed between 1935 and 2003 (99, 100).

**Year of development**	**Antimicrobial classes**
1935	Sulphonamides
1941	Penicillins
1944	Aminoglycosides
1945	Cephalosporins
1949	Chloramphenicol
1950	Tetracyclines
1952	Macrolides/Lincosamides/Streptogramins
1956	Glycopeptides
1957	Rifamycins
1959	Nitroimidazoles
1962	Quinolones
1968	Trimethoprim
2000	Oxazolidinones
2003	Lipopeptides

### Drug Development in This Century

The early part of the 21st century saw the development of relatively few antimicrobial drugs, (26, 101, 102), and most newly introduced drugs had limited application.

Some antimicrobials were developed from natural resources. Healthcare in developing countries benefitted from economical, often soil-derived, medicines. When their potential had been exploited to the point of exhaustion, there was a need to synthesize new antimicrobial molecules (37, 103–105). However, research into developing synthetic antimicrobial molecules from existing natural medicines had poor success rates (103, 106).

Global research endeavor focused on developing new approaches to overcome MDR. Early this century, infection caused by antibiotic-resistant microbes, both by gram-negative bacilli (GNB) and gram-positive pathogens such as *Enterococcus faecium, Staphylococcus aureus, Klebsiella pneumoniae, Acinetobacter baumannii, Pseudomonas aeruginosa, and Enterobacter species* had significantly increased human morbidity, mortality, hospitalization, and augmented hospital stay and healthcare costs throughout the globe (107–113). Subsequent research used highly advanced technology to explore “genomics and combinational chemistry” but was often unsuccessful in finding new molecules with the necessary efficient antimicrobial activity (114). Box 1 lists some antimicrobials introduced in the last 20 years.

Box 1 **Antimicrobials introduced 2003 to 2013.**

**Daptomycin** is a lipopeptide antibiotic introduced in 2003 for the treatment of systemic and life-threatening infections caused by Gram-positive organisms. It is a naturally occurring compound found in the soil saprotroph *Streptomyces roseosporus* (115).

**Tigecycline**, introduced in 2005, is an intravenous glycylcycline (tetracycline derivative) antibiotic. Active against both Gram-positive and Gram-negative organisms, it was intruded to combat MDR organisms, but has since encountered drug resistance (116, 117).

**Retapamulin** is a topical antibiotic introduced in 2007. It is used in the treatment of bacterial skin infections such as impetigo, and it is the first drug in the new class of pleuromutilin antibiotics to be approved for human use (118, 119).

**Fidaxomicin**, FDA licensed in 2011, is the first member of a class of narrow spectrum macrocyclic antibiotic drugs called tiacumicins. It is a fermentation product obtained from a natural actinomycete. Currently very expensive, it is used for the treatment of *Clostridioides* (*Clostridium*) *difficile* infection (120–123).

**Bedaquiline**, approved by the FDA in 2012, is an oral preparation used to treat multi-drug-resistant tuberculosis (MDR-TB) in combination with other anti-tubercular medication. It is a diarylquinoline, and blocks ability of *M. tuberculosis* to make adenosine 5'-triphosphate (ATP) (124).

**Televancin** is a synthetic derivative of vancomycin, originally used in skin infections. Subsequently it has been found useful in hospital acquired *Staphylococcus Aureus* pneumonia, for which it was licensed in 2013 (125, 126).

#### New Combinations of Antimicrobials

Some promising new combination drugs have been introduced since 2015, They are mostly expensive and their restricted indications have the effect of limiting their prescription to a small number of specialists in developed countries.

Ceftazidime-Avibactam combination was first licensed by the FDA in 2015 (127). Ceftazidime, a third-generation, antipseudomonal cephalosporin antimicrobial with an advantageous pharmacokinetic profile, and avibactam, a next-generation, β-lactamase inhibitor. This combination was approved for the treatment of complicated intra-abdominal infections, complicated urinary tract infections, hospital-acquired bacterial pneumonia, and ventilator-associated bacterial pneumonia (128, 129). Ceftazidime has an advantageous pharmacokinetic profile (130–132) and Avibactam is a microbial enzyme inhibitor designed to overcome the key issue of cephalosporin resistance. The clinical importance of avibactam is its ability to inhibit some carbapenemases, thereby giving a therapeutic option for some infections caused by MDR organisms when there are no other options (133, 134). Sadly, although this combination offers major advantages in the management of complex infections, multiple studies have described the emergence of resistance among carbapenemase-producing *Klebsiella pneumoniae* (CPKP) pathogens (135–140).

The US FDA also approved the three-drug combination imipenem-cilastatin/relebactam for hospital-acquired bacterial pneumonia and ventilator-associated bacterial pneumonia in adults in 2019 (141). The indications for this combination agent are the treatment of complicated urinary tract infections and complicated intra-abdominal infections (142). The combination of imipenem-cilastatin has been in clinical use since 1985 (143). The addition of relebactam, a novel beta-lactamase inhibitor in the diazabicyclooctane class, was the innovation in this combination (141, 142), conferring advantage by overcoming the resistance issue of several imipenem-resistant microorganisms, including *Enterobacteriaceae* and *Pseudomonas aeruginosa* (144). Relebactam impedes bacterial synthesis of class A and C β-lactamases, consequently, lowering the minimum inhibitory concentration (MIC) and improving imipenem sensitivity activity against *P aeruginosa* (144). The approval of imipenem-cilastatin/relebactam brings new hope for enduring combat against MDR pathogenic microbes (144–146).

#### New Single Agents

In 2018 the FDA approved plazomicin for the management of very severe chronic urinary tract infections (CUTI) including pyelonephritis in adult patients (147). In licensing plazomicin, the regulatory authority imposed an important restriction by limiting plazomicin prescription only to those hospitals using trough-based therapeutic drug management (TDM) systems to minimize the possibility of renal adverse effects among recipient patients. This important restriction to protect highly vulnerable patients should reduce irresponsible prescription. Plazomicin is a newly developed aminoglycoside antimicrobial which inhibits microbial protein synthesis by binding with the microbial 30S ribosomal subunit in a concentration-dependent manner. Plazomicin exhibits a broad spectrum of activity against aerobic gram-negative pathogens including extended-spectrum β-lactamase–producing (ESBL) *Enterobacteriaceae*, CRE, and microorganisms capable of producing aminoglycoside-modifying enzymes (148, 149). Plazomicin resistant to those pathogens are encoded with 16S ribosomal RNA methyl-transferases (16S-RMTases) especially for *Enterobacteriaceae* spp (150–154). It has, however, been reported that the 16S-RMTase enzyme can bind with 16S rRNA target sites in the presence of plazomicin as with other aminoglycosides. So far 10 different type of acquired 16S-RMTases have been identified among gram-negative pathogenic microbes. The majority of 16S-RMTases were post- transcriptionally methylate residue G1405 of 16S rRNA; consequentially, these pathogens can already develop the same high levels of resistance to plazomicin as to other aminoglycosides (gentamicin, tobramycin, and amikacin) (154).

Cefiderocol is a parenteral siderophore (from the Greek meaning iron carrier). Low molecular weight Fe^3+^ chelating molecules are synthesized by microbes such as bacteria and fungi and serve primarily to transport iron across cell membranes (155). This novel cephalosporin, developed in Japan, (156) was FDA approved in 2019 (157). Cefiderocol is a β-lactam antimicrobial, exerting its principal mechanism of action through inhibition of the cell wall synthesis of Gram-negative pathogens (158). It possesses a similar mode of action topenicillin—the first antibiotic. The novelty of cefiderocol lies in its ability to enter the microbial periplasmic space. It is too early to tell how effective the siderophore qualities will be in countering β-lactamase drug resistance (159).

## Scientific and Economic Challenges for Antimicrobial Development

If the flow of antimicrobials through the pipeline of development seems to have slowed, (102, 160) one suggested reason is that giant pharmaceutical companies have deprioritized funding antibiotics research, because the development of medications utilized in lifestyle-related sicknesses generates much more substantial financial gain (53, 99, 100). The invention of a new antimicrobial agent or a new class of antimicrobials in the last 35 years has been difficult, time-consuming, and problematic because of many issues (101, 161). In addition, it is taking longer for new antimicrobials to obtain approval from drug regulatory bodies, and hence new agents are coming onto the clinical market too slowly and too infrequently to combat current alarming levels of AMR (162, 163). Despite pharmaceutical industry and academic institute sponsored research, the development of innovative antimicrobial agents for clinical use has dwindled.

The principal approaches to combating AMR remain in two significant areas, Firstly, the accurate identification of molecular targets that are not predisposed to quick development resistance mechanisms is a priority (101, 162). Secondly, in order to overcome current limitations on treatment of contagious diseases, there is an urgent need to move past the current well-known five principal core mechanisms of antimicrobial action (i.e., cell wall synthesis, protein synthesis, RNA polymerase and DNA gyrase, folate mechanism, and membrane structure).

One particular ambition is to develop novel antimicrobial molecules to overcome the ability of gram-negative microorganisms to develop efflux-mediated drug resistance, and molecular target alteration (101, 162, 164, 165). Although pharmaceutical industries have in the past benefitted considerably from developing innovative antimicrobial agents for the management of contagious diseases; nevertheless, many have discontinued their antimicrobial research investment since the late 1990s. Not only has further innovation of new antimicrobial molecules became extreme difficult, but also the profit margin for this activity turns out to be very low in proportion to the enormous investment required (106, 161, 166–168).

Profitability is affected by the speed with which microbes develop resistance to newly introduced antimicrobials, thereby severely restricting sales of the drug (161, 166, 169, 170). Additionally, the significant difference between antimicrobials and other medicines for chronic non-communicable disease (NCDs) management is that antimicrobials are taken for a short period to cure infectious diseases. In comparison, medicines for NCDs like hypertension, diabetes mellitus, bronchial asthma, hyperlipidemia, mood disorders often need to be prescribed over the patient's lifetime (161). The enormous investment required for drug development is a powerful consideration for profit-focused pharmaceutical companies (161, 171–173). Some major transnational pharmaceutical industries have lost interest in investing in antibiotic research as antimicrobials became less profitable. Dr Mahesh Patel has drawn attention to a particular problem arising from the closure of multiple antibiotic discovery programs witnessed over past twenty plus years, it has led to crippling loss of skill sets specifically required for the discovery of antibacterial drugs. Given such scenario, large pharma companies are now finding it difficult to assemble a full multidisciplinary team, such as is required for this type of productive research program. “Society wants a cheaper antibiotic, but the cost of development will be high. So, we need to manage these two conflicting needs.” (174).

Multiple studies attest to the continuing low pace of development of new antimicrobials (66–80). The causes, which are not limited to corporate-driven planning of pharmaceutical industries, also include the guidelines, policies, and processes of supervisory bodies responsible for medicine approval, and regulation. Perhaps the best-known of these bodies is the USA's Food and Drug Administration (FDA),

Changes which the FDA has made to their guidelines on research protocols over the past 25 years have resulted in some restriction of the ongoing work by scientists to develop new antimicrobials (161, 175, 176). Under the FDA's current guidelines placebo-controlled clinical trials for antimicrobials are deemed to be detrimental to human rights. The current FDA protocol for antimicrobials research necessitates large sample sizes and, as a result, hugely increases the financial overheads. Thus, the commercial incentives for the pharmaceutical industry to invest in antimicrobial research, including the quest to overcome drug-resistant microorganisms, have been drastically reduced (175, 176).

Despite all the disincentives new antimicrobial drug innovation remains a significant thrust for scientists in drug-developing industries (161). The pharma-industries adopted genomics and target-based screening technologies in the 1980s to enable and accelerate the antimicrobial drug innovation procedure (161, 177–179). Undoubtedly, novel and efficient biochemical and genomic target-based techniques for screening approaches to human drug development have been a significant advance for (106), Nonetheless, little has yet emerged from the use of these modern technologies to increase optimism about development of novel antimicrobials to combat AMR infectious diseases in the near future (180–182). At the present time we can only speculate about whether the global drive to develop vaccines and antiviral compounds for the Covid-19 pandemic could have positive spin-offs for antimicrobials.

## Antimicrobial Resistance Around the Globe

Microbial resistance to antibiotics has been a predictable to the point of certainty. The inescapable emergence of the resistance has been observed from the initial days of the antibiotic research and clinical utilization. Nevertheless, the advent of the most notorious resistant microbial strains in the last 25 years has transformed the situation into a life-threatening issue (168). AMR is a tremendous public health problem all over the globe. There is universal distress as the threat to human life rises (183). AMR pathogenic microorganisms have emerged, both in humans and in animals on each of the earth's continents. They have been identified in arctic regions and even on the international space station (184–186). Consequently, infectious diseases remain the most important cause of mortality (187). A brief timeline of the antimicrobials' development and resistance (81, 130–132, 188–210) is depicted in Table 2. Multiple research reported by the early-1940s presence penicillin inactivator enzyme among *Staphylococcus aureus;* thereby, penicillin was resistant (211–213). Abraham and Chain revealed that published in 1940 that an *Escherichia coli* was capable of inactivating penicillin by producing penicillinase enzyme (213, 214).

**Table 2:** Timeline of antimicrobials development and resistance (81, 130–132, 188–210).

**Antimicrobials**	**Year**		**The resistant pathogen**
	**FIRST marketed for clinical use**	**First reported resistance**	
Penicillin	1943	1940	*Escherichia coli*
Penicillin G	1942	1942	*Staphylococcus aureus*
Streptomycin	1945	1952	*Mycobacterium tuberculosis*
Chloramphenicol	1949	1973	*Streptococcus pneumoniae*
Tetracycline	1950	1959	*Shigella flexneri*
Erythromycin	1953	1968	*Streptococcus pyogenes*
Isoniazid	1954	1992	*Mycobacterium tuberculosis*
Colistin	1959	1999	*Acinetobacter baumannii*
Methicillin	1960	1962	*Staphylococcus aureus*
Ampicillin	1961	1972	*Haemophilus influenzae*
Gentamicin	1967	1979	*Enterococcus*
Rifampin*	1968	1985	*Mycobacterium tuberculosis*
Vancomycin	1972	1988	*Enterococcus faecium*
Ceftriaxone	1982	2009	*Neisseria gonorrhoeae*
Imipenem	1985	1998	*Enterobacteriaceae*
Ceftazidime	1985	1987	*Enterobacteriaceae*
Ciprofloxacin	1987	1993	*Salmonella typhimurium*
Imipenem	1987	2001	*Klebsiella pneumoniae*
Meropenem	1996	1998	*Acinetobacter species*
Levofloxacin	1996	1996	*Streptococcus pneumoniae*
Linezolid	2000	2000	XDR Tuberculosis
		2001	*Staphylococcus aureus*
Daptomycin	2003	2005	Methicillin-Resistant *Staphylococcus aureus*
Ceftaroline	2010	2011	*Staphylococcus aureus*

Currently, almost all clinical used antimicrobials, including colistin, the final weapon in the armament safeguarding humans from deadly gram-negative infections, have also developed resistance (168, 215–217). Furthermore, internationally, the threat to public health from the exponential progression of MDR, XDR, and PDR gram-negative microorganisms has become critical.

Although widespread use of any antibiotics inevitably promotes resistance in micro-organisms over time, many antibiotics have given good service worldwide for 25 years or more before they become redundant. After a period of disuse, sometimes brought about as much because of adverse effects as ineffectiveness due to resistance, desperate attempts have been made to bring back into use some age-old antibiotics with a new dosage schedule. These drugs had previously been dropped from clinical use because of their profound adverse effects. Unsurprisingly, resistance has also speedily arisen to these resurrected antimicrobials (218).

Besides the list shown in Table 2, PDR was reported in the year 2004/2005 for Acinetobacter and Pseudomonas, and in 2009 for Enterobacteriaceae. Rifampicin mono-resistant tuberculosis (RMR-TB)is rare globally. In 2014, The World Health Organization (WHO) appraised that only 1.1% of TB patients around the planet had rifampicin resistance without resistance to isoniazid (197, 198, 210).

## Current Most Dangerous Pathogenic Microbes

In 2017 WHO first published the list of antimicrobial-resistant (Table 3) “priority pathogens” —a directory of 12 families of microbes that pose the most extreme public health risks around the globe (219). In 2019 the Center for Disease Control and Prevention (CDC) classified 18 antimicrobial-resistant bacteria and fungi into three classes by level of public health issues of urgency, severity, morbidity, and mortality (Table 4) (220).

**Table 3:** WHO *priority list: infectious micro-organisms for research and development of novel antimicro*bials.

**Name of the Pathogens**	**Name of Antimicrobial Resistance**
**PRIORITY 1: CRITICAL**	
*Acinetobacter baumannii*	Carbapenem
*Pseudomonas aeruginosa*	Carbapenem
*Enterobacteriaceae*	Carbapenem [Extended Spectrum B-Lactamase (ESBL) Producing]
**Priority 2: HIGH**	
*Enterococcus faeciu*m	Vancomycin
Methicillin-Resistant *Staphylococcus aureus*	Vancomycin
*Helicobacter pylori*	Clarithromycin
*Campylobacte*r spp.	Fluoroquinolone
*Salmonellae*	Fluoroquinolone
*Neisseria gonorrhoeae*	Cephalosporin and Fluoroquinolone
**Priority 3: MEDIUM**	
*Streptococcus pneumoniae*	Penicillin
*Haemophilus influenzae*	Ampicillin
*Shigella* spp.	Fluoroquinolone

**Table 4:** CDC threats to public health.

**Urgent threats**	**Serious threats**	**Concerning threats**
Carbapenem-resistant *Acinetobacter*	Drug-resistant *Campylobacter*	Erythromycin-resistant group A *Streptococcus*
*Candida auris*	Drug-resistant *Candida*	Clindamycin-resistant Group B *Streptococcus*
*Clostridioides difficile*	ESBL-producing *Enterobacteriaceae*	**Watch Lists**
Carbapenem-resistant *Enterobacteriaceae* (CRE)	Vancomycin-resistant *Enterococci* (VRE)	Azole-resistant *Aspergillus fumigatus*
Drug-resistant *Neisseria gonorrhoeae*	Multidrug-resistant *Pseudomonas aeruginosa*	Drug-resistant *Mycoplasma genitalium*
	Drug-resistant non-typhoidal *Salmonella*	Drug-resistant *Bordetella pertussis*
	Drug-resistant *Salmonella* serotype Typhi	
	Drug-resistant *Shigella*	
	Methicillin-resistant *Staphylococcus aureus* (MRSA)	
	Drug-resistant *Streptococcus pneumoniae*	
	Drug-resistant Tuberculosis	

One estimate is that development of a new antimicrobials molecule takes some 15–20 years (221, 222) and financial support of over US$ 2.6 billion (223). Another study estimated this at a minimum of 10-years and needs over £(GBP)1.5 billion (224). Most long-standing groups of antimicrobials have considerable resistance problems, and new approaches are needed (50).

The principal mechanisms of resistance are: restraining uptake of an antimicrobial; alteration of an antimicrobial target; degradation of an antimicrobial; and active efflux of an antimicrobial. These mechanisms can appear as innate to the micro-organism, or transferred from other micro-organisms (216, 225). Antimicrobial resistance, conferring enhanced survival for a rapidly replicating microbial species, is passed down genetically as the species evolves. Microbes employ two chief genetic plans to mitigate the antimicrobial line of attack. These are either mutations in gene(s) frequently correlated with the mode of action of the antimicrobial compound, or getting hold of foreign DNA coding for resistance factors through horizontal gene transfer (HGT) (216). Typically, microbes gain external genetic elements in one of three ways: transformation; transduction; or conjugation (216, 226).

Plasmids are extrachromosomal DNA molecules that can be found throughout the microbial population, as in other realms of life. The plasmid is considered as a means of transportation for rapid modification and adjustment of microbial populaces to altering ecological settings. Plasmid-arbitrated gene transfers act as the central task, not only in the deployment and propagation of antimicrobial resistance genes, but also in the forming of degradative pathways and pathogenicity determining factors of pathogenic micro-organisms (227, 228). Horizontal gene transfer (HGT) contributes to the rapid dissemination resistance of microbial genes. The dynamic forces of gene transmission that confer antimicrobial resistance are yet to be fully defined. There are manifold HGT mechanisms by which genes are liberated from their normal vertical microbial genetic legacy. These include conjugation by plasmids, transduction by bacteriophages, and natural alteration and conversion by extracellular DNA elements that permit genetic molecules to leap among strains and species. (Consequently, HGT enables multiple unrelated pathogenic micro-organisms to act as agents of an epidemic by transferring drug resistance conferred by an antimicrobial resistance gene (ARG) (229).

## Rational use of Medicine and Irrational Prescribing OF Antimicrobials

### The Situation in Developed Countries

The Greek physician and earliest anatomist Herophilus [335-280 BC] mentioned that “medicines are nothing in themselves but are the very hands of God if employed with reason and prudence,” indicating that the notion of the rational use of medicines is a few thousand years old (230). The judicious or rational use of medicine (RUM) was defined by WHO in 1985 as “Patients receive medications appropriate to their clinical needs, in doses that meet their requirements, for an adequate period, and at the lowest cost to them and their community” (231). The World Bank has also defined RUM in two tiers: (i) the utilization of medicines based on scientific data regarding drug efficacy, safety, and compliance; and (ii) the need to maximize the benefit of medicine in the resource-limited health system to ensure cost-efficacy (232, 233). RUM has been considered as the strategic issue in providing effective and quality healthcare (234, 235).

In the developed world antimicrobials are almost without exception legally defined as prescription-only medicines. However, a recent Norwegian study has shown how frequently travelers are able to buy antibiotics over the counter, even in EU countries (236).

Awareness of the consequences of irrational and imprudent prescriptions in raising utilization of antimicrobials with a consequential higher rate of AMR (237, 238), has led to many initiatives to make antimicrobial prescribing more careful and selective (239–241). Careful, rational prescribing, can be seen as a quality marker for healthcare (242).

However, until recently, disappointingly high rates of irrational prescribing were still seen in developed countries around the world. For example, in the USA, despite tight regulation by the FDA, rates as high as 30% and in China, where the reputable National Medical Products Administration (NMPA) is the agency responsible for strict drug strategies (243, 244) non-concordant prescribing up to 60% has been reported (237, 238, 245, 246). Even strong regulation, such as that in Europe, seems to fall short of preventing health professionals from prescribing antimicrobials imprudently (169, 247–250), which is why there have been recent initiatives in many developed countries to raise public awareness of the issues (thereby reducing pressure on prescribers) and to educate the prescribers better about AMR (251–253). Public awareness is important to tackle problems such as self-medication using leftover antibiotics from the previous prescription, and optimal duration of prescriptions is an example of health professional practice amenable to relevant education among the healthcare providers themselves (254).

### The Situation in Developing Countries

Although the sale of antibiotics without prescription is illegal in all European Union (EU) countries, in practice they are available over the counter in many EU countries (255–257). This is also the case in several low and middle-income countries (LMICs), despite laws requiring that antimicrobials must only be dispensed on the prescription of registered medical practitioners (253, 258, 259). It has been reported that in the majority of LMICs, at least 19–100% of all antimicrobials are retailed without a prescription outside northern Europe and North America because of the loosely regulated and implemented private segments of healthcare (260). Furthermore, the WHO recorded that roughly 80% of all prescribed pharmaceutical products in LMICs are given out by a person who possesses almost no training or educational background in dispensing medicine (261–264).

The regulatory agencies in many LMICs failed to implement the necessary laws regarding prescription-only medicine all over the country (265, 266). Consequently, pharmacies take the opportunity to maximize their profit by selling antibiotics over the counter and over the internet (265, 267, 268). It is unsurprising that patients themselves opt to buy antibiotics and other prescription-only medicine to reduce the cost of treatment and the hassle of obtaining treatment either through general practitioners or public and private outpatient services (269, 270).

A study conducted in Iran revealed that at least 42.7% of antibiotic prescribing was not done rationally. The authors advocated the development of new programs in collaboration with WHO to prevent the imprudent use of antimicrobials (271). Indeed, the problem is global. For example, multiple studies supported the finding that the rate of irrationally prescribed antimicrobials was even higher in Pakistan, at the rate of 60.3% (272, 273).

Even governmental attempts to improve the situation can be thwarted. For example, the Tanzanian government became highly concerned about the irrational prescribing of antibiotics and AMR (274, 275). They developed a regulatory policy that antimicrobials be limited to prescription-only medicines, sold only in drugstores with accredited drug dispensing outlets (ADDOs), and named in the local area Duka la Dawa Muhimu (DLDM). Moreover, all antibiotics are sold under the strict supervision of registered pharmacists, who are specially trained for antimicrobials dispensing. Regardless of such strict policies, the irrational utilization of these medicines remains at a very high rate of 80–85% (274–277). Additionally, 88.8% of antibiotic procurements were found to be sold over the counter for self-medication with antibiotics (278). In South Africa, more than 54% of antibiotic prescribing violates the national guidelines for prescribing antimicrobials. Ignoring guidelines results in the use of antibiotics for non-indicated diseases such as the common cold or viral infection (21.6%), inappropriate dosing (12.9%), inappropriate medicine (11.5%), and inappropriate duration of therapy (9.5%) (279). The reported use of antimicrobials in Egypt during the years 2000–2015 was extremely high—always one of the top three for per-capita antibiotic consumption worldwide. Yet multiple Egyptian studies reported a high rate of antimicrobials consumed without scientific reasons or guidelines (280–283). Additionally, antimicrobials dispensing standard in Egypt does not meet the national guideline in both qualities and quantities, which ultimately results in increasing drug resistance and the loss of healthcare budget (279, 280).

The Latin American nations, including Brazil, are troubled by irrational consumption of antimicrobials, especially self-medication with antibiotics. Even though Brazilian medicine-related law does not permit self-medication with antibiotics, around one-fifth of their antibiotic total use was self-medicated (284). Another recent report shows a much higher rate of self-medication with antibiotics at 66.2%, with around 33% of patients consumed antibiotics without any microbial diseases (284, 285). In 2010 the Brazilian Health Surveillance Agency developed a new policy for antibiotic sales over the counter without a prescription. This necessitates that pharmacists maintain a register of antibiotic sales with genuine evidence of the pharmacist's antimicrobials prescriptions to be shown to the public audit team during routine and extraordinary inspection (286, 287). This new law asserted that if any pharmacists fail to show such evidence, they face stringent civil and criminal charges. One study regarding the implementation of such laws found a decrease in the number of antimicrobials without prescription in Brazil. Consequently, studies concluded that stringent policy and planning implemented by the regulatory authority could change the overall situation of drug consumption by promoting rational and prudent utilization of antibiotics and other medicine (286, 287).

## Antimicrobial Prescribing: Knowledge, Attitudes, and Practice (KAP)

In most of the developed world and many developing countries, the prescribing of antibiotics is guided by local and national formularies (288, 289). Most of these deploy step wise guidance in terms most infections, escalating antibiotic therapy by counseling first-, second-, and third-line antibiotics for each condition. Formulary developers take into account a number of relevant issues including cost and availability; the site and severity of the infection; and the likely organisms. Severe life-threatening infections may need to be treated “blind” if the patient's life is to be saved and so “the big guns” of the antibacterial armamentarium are often reserved for these situations. Their expense, and perhaps the need for intravenous administration, are factors which often limit their use to specialist hospital units, and their relatively infrequent use may help to limit the development of microbial resistance. The more local the formulary, the easier it is to take into account local epidemiology and resistance trends, especially when these formularies are accessed on-line. It is still down to the individual prescriber to take account of factors such as the severity of infection, patient's age, co-morbidities, and immune status.

Sound principles of antibiotic stewardship are vital in guiding physicians responsible for prescribing the appropriate antibiotic at the correct dose and for the right duration. The same stewardship principles should enable prescribers to resist prescribing an antibiotic at all, if it is inappropriate to do so, but this is one of the most difficult issues. Saying “No” to a patient pressing for treatment requires advanced skills in interpersonal communication, particularly in situations where the patient is likely to go elsewhere if they do not like what the doctor is saying.

Where treatment is needed, prescribers must be mindful of the importance of achieving sustained pathogen eradication, leading to clinical cure. This is all the more important when dealing with serious infection where the objective is to minimize relapse. In hospital practice, judicious antimicrobial prescribing requires a dosing regimen which both ensures patient's safety from adverse effects and facilitates early discharge from ICU or hospital.

Prescribers are often faced with complex situations where there is a dearth of guidance. Clinical trials have often not systematically evaluated an antimicrobial for the predicament of the individual patient concerned, and it is down to the prescriber to use best judgement. For prescribers, knowledge about drugs is vital, but so too is an attitude that promotes the responsible behaviors to look after both the individual patient and the wider community, and the skills to support best practice.

### Antimicrobial Prescribing KAP Among Medical Practitioners

Studies across the world demonstrate that there are serious KAP deficiencies amongst prescribers. We select here four examples: one each from Malaysia, India, China, and Baltimore USA.

The Malaysian study, conducted in a public hospital, looked primarily at knowledge. Medical officers scored moderately well on knowledge, reflecting the fact that 62.0% of the research respondents expressed confidence in prescribing antibiotics. However, despite their lack of confidence, only 18% of the respondents actually discussed the therapeutic options with a colleague before prescribing antibiotics or other medicines (290).

The Indian study conducted in tertiary hospitals revealed that was statistically significantly related to the periodic updates on bacterial resistance patterns improved medical doctors' knowledge levels, as did internal and external antibiotic-related courses. More senior doctors with 11 years' average experience were more likely to have a scientific and rational basis for antimicrobial utilization. The study also highlighted that 40% of the study respondents reported that their institute did not have a formulary or an infection control policy (291).

A recent Chinese study reported that medical doctors possessed limited knowledge and skills about prescribing antibiotics with, on average, respondents answering correctly just over half of the questions to assess their understanding (292). The study reported that medical doctors were anxious about AMR and believed that resistance is promoted by “overprescribing.” They claimed to usually avoid patient pressure for antibiotic prescribing and to practice “defensive prescribing” policy, being aware of the impact of overprescribing on AMR. This study noted, however. that physicians were poorly motivated to improve and upgrade prescribing quality. The structural equation modeling (SEM) used in this study showed that poor knowledge, obliviousness to AMR, and low enthusiasm to upgrade prescribing quality all correlated with overprescribing practice for antibiotics (*p* < 0.001) (292).

The study conducted at Johns Hopkins Hospital, a 1,000-bed university teaching hospital in Baltimore, revealed that residents in medicine had a significantly higher mean score for antimicrobial prescribing than those in surgery, emergency medicine, and obstetrics and gynecology (*p* = 0.04). There were, however, no statistically significant variations in total scores between the four groups when equated for years of training, and this remained the case for sub-scores on both elementary and critical-thinking issues. At this hospital, the junior doctors were found to have less than the desired knowledge level about antibiotics, and their knowledge did not improve significantly after attending a training program (293).

### Educating Medical Students About Antimicrobial Prescribing and Resistance

A study conducted in the United Arab Emirates compared KAP for medical students with their non-medical student colleagues. Unsurprisingly, this study revealed that medical students had statistically significantly higher KAP regarding antibiotic use than non-medical students with *p* < 0.001 for knowledge and attitude and *p* = 0.002 for practice (294). Medical student performance improved over time on the course, but worryingly a third of all medical students were confused about whether antibiotics were effective against bacteria or viruses.

There were some interesting findings in a Chinese study which also reported that the knowledge scores of medical students improved as they progressed across their course, and were statistically significantly higher than that of non-medical students regarding the prudent use of antibiotics (*p* < 0.001). However, the medical students' own consumption level of antibiotics was also statistically significantly higher than that of non-medical students (*p* < 0.001) (295). What this study also reported was disparity between knowledge and personal practice as regards self-medication. Senior medical students consumed antibiotics irrationally, indicating that the medical curriculum was not managing to promote prudent consumption of antibiotics in the future prescribers (295).

This appears not to be an isolated problem. Another Chinese multicenter study revealed that among medical students who had self-limiting diseases in the last month, 54% of them were self-medicated, and 27% took antibiotics. Only 21% of them consulted local medical doctors, among whom, 58% were prescribed antibiotics. Another study found that the KAP scores regarding antibiotics were significantly lower among those who practiced self-medication with antibiotics, used antibiotics for prophylaxis or requested antibiotics from their physician, compared to those with no self-medication with antibiotics (296).

Studies from India also address the relationship between what the medical student knows and what he/she does. One Indian study reported that clinical medical students were more aware of issues around self-medication with antibiotics than preclinical students. Knowledge levels reported in another Indian study were “good” but the attitude and practice scores were quite low by comparison (297). However, another Indian study reporting high scores in the antibiotics and resistance knowledge domain found that 67%, of the research respondents practiced self-medication with antibiotics, 70% stocked left-over antimicrobials, and 42% consumed these left-over medicines (298).

One Pakistani comparative study reported that medical students' knowledge scores were statistically significantly lower than the pharmacy students (*p* < 0.05). Most of the study respondents (79%) from both medical and pharmacy students reported that antimicrobials were over-prescribed in Pakistan (299).

## Overcoming The Problems

### Undergraduate Medical Education and Training on Antimicrobials

“*Prescribing is one of the commonest tasks expected of new doctors and is a complex process involving a mixture of knowledge, judgment, and skills* (300).”

Rational prescribing is a critically important attribute for all doctors. (301, 302). Prudent prescribing practice necessitates a comprehensive mastery of the clinical pharmacology and therapeutics, ability to work from first principles in unfamiliar, complex, or ambiguous situations and high quality decision-making skills, honed by experience (300).

Since clinical pharmacology and therapeutics form the scientific basis of prudent prescribing (303–306), it is imperative to allocate adequate priority to these subjects in both undergraduate and postgraduate curricula, and to ensure meaningful assessment of real-life prescribing skills (300, 303, 304, 306–309).

There seems to be a strong impetus for improving undergraduate education, arising from concerns around the world about the levels of skills of newly qualified doctors to prescribe safely and effectively medicine. One British study in 2009 revealed a lack of uniformity in teaching clinical pharmacology and therapeutics and deficiencies in the teaching and assessment of prescribing skills of medical students in most British medical schools (307). Two years later, a study of newly qualified doctors, trained in very different medical schools, found that graduates entering their first postgraduate foundation year (F1) felt under-prepared for prescribing. However, there was improvement over the F1 year through practical experience and support. Participants reported that learning in an applied setting would be helpful and increase confidence in prescribing. No clear differences were found in preparedness to prescribe between graduates of the three medical schools. The authors recommended increasing the number of opportunities at medical school to develop the skill-based, applied aspects of prescribing in a controlled, “real” environment. They suggested that either simulated activity could provide this experience to undergraduates or, on clinical placements, they could write prescriptions and drug charts to be checked and signed by a doctor (310).

Another study involving 185 medical schools in 27 countries of the European Union (EU) found significant variation in clinical pharmacology and therapeutics coursework and little or no provision for medical students to be exposed to real-world clinical settings for them to develop their prescribing skills. Researchers highlighted the need for collective effort to improve undergraduate clinical pharmacology and therapeutics education congruently (308).

In a cross-sectional study of 296 medical schools in 29 EU countries, students' perception of a need for more educational interventions to support learning about rational antibiotic use varied between 20.3% (Sweden) and 94.3% (Slovakia). Lower scores in clinical pharmacology and therapeutics education and higher self-reported perception for more clinical pharmacology and therapeutics course work were associated with prescribing antibiotics for non-susceptible microbes (*p* < 0.001) (311).

Likewise, another study found that French medical students demanded more educational training in prescribing than Swedish counterparts, and the final year French medical students' self-perceived readiness to prescribe an antibiotic after graduation was lower than their Swedish counterparts (312).

A number of other studies have also supported the need for educational interventions to combat widespread findings of imprudent and irrational prescribing of antibiotics (239, 313–316).

As well as increasing the curriculum time allocated to teaching and learning about the teaching of proper selection of antibiotics (315) the emergent message from research studies is to promote educational inventions in real-life settings for undergraduate medical students (239, 310, 313).

### Strategies to Improve Antibiotic Prescribing, and Reduce Self-Medication

Irrational consumption of medicines has very deleterious consequences for patients and healthcare systems, both budgetary and societal (153). WHO classifies irrational prescribing as a chronic progressive disease of any healthcare system, and as in any other chronic diseases, treatment is challenging and cure elusive, but prevention is possible (317, 318). Strategies to combat irrational prescribing include educational, managerial, regulatory, and economic interventions (153, 318, 319).

#### Postgraduate Educational Strategies

Undergraduate leaning has been covered above. Similar learning opportunities are needed for postgraduates. As they progress through advanced training, the science of clinical pharmacology and therapeutics needs revisiting to support doctors becoming responsible for prescribing more specialized antimicrobials. They need training to be able to assess the appropriate prescribing of new clinical entities, and it is vital that their prescribing is audited and that the doctor is given appropriate feedback. Multiple systematic reviews conclude that positive improvements in prescribing competency are most likely to be achieved with educational inventions specially tailored to local needs. (316, 320–322).

#### Managerial Strategies

Managerial strategies include: “Essential drug lists, kit system distribution, re-printed order forms, stock control, course-of-therapy packaging, and effective package labeling” (323). A systematic review of managerial interventions for antibiotic prescribing in LMICs improved correct dose-taking by 8.0% and adherence to the treatment plan by 54.0% as well as a 29.1% mean improvement in rational prescribing antibiotics for the treatment of resistant microbial infection and a 54.8% mean improvement in prophylactic use of antibiotics (324).

#### Regulatory Strategies

Poorly functioning National Medicines Regulatory Agencies (NMRAs) contribute to irrational medicine utilization and prescribing. Additionally, the absence of a national health policy on antimicrobial prescribing, the absence of drug formularies or appropriate dispensing guidance, and sagging control over how medicines are promoted to prescribers and consumers have all been found to underpin irrational medicine practice (254, 325–330). WHO estimates that only 30% of the world's NMRAs perform their principal supervisory and monitory tasks (324, 331).

WHO describes how regulatory agencies should limit the open choices to health professionals prescribing medication. NMRA approaches consist of strict rules, prioritizing generic medicines, promotion of essential medicines, and taking doubtful medications off the market (331, 332). These stringent policies are often poorly accepted by both patients and health professionals. An untoward consequence can, therefore, be the promotion of imprudent prescribing and utilization of medicine (331). Even when controls are in place, they are often circumvented or overcome by consumer pressure on prescribers and dispensers. There is a risk that people who are adamant that they want an antibiotic will desert the doctor who declines to prescribe it. The challenge for regulators is to both control the prescribing and simultaneously undertake effective public health messaging. Regulation to prevent the sale of antibiotics without prescription is essential.

Another regulatory strategy is to focus significant effort on the single most important disease area. For many countries this is acute respiratory tract infections (ARTIs). WHO estimates that <30–40% of healthcare providers issue or enforce national or international treatment guidelines on ARTI's (333). Around 60% of patients were given antimicrobials in ARTIs. Their report comprises of 679 research studies in 97 LMIC nations of (333). Other studies provide further evidence of how many NMRA's exist in LMICs, on many occasions, are not managing to control and promote rational and prudent prescribing (153, 318, 334–336), so there is scope for very significant improvement here.

#### Economic Strategies

Downward pressures on prescribing from regulators can have economic benefits. In Chile, a new regulatory policy, launched in 2000, successfully decreased antimicrobials sales in the private health-sector from US$37.6M 1996 to US$32M in 2000 (326).

Other economic strategies to prevent irrational prescribing are include the abolition of all commercial incentives to those involved in prescribing antibiotics and other medicines. This includes preventing direct monetary inducements or other forms of financial motivational benefits being offered to healthcare staff at any level (153, 325, 332, 337–341). Multiple studies report that the concept of “essential drugs,” generic drug utilization programmes, and robust implementation of national guidelines for treatment can bring about significant benefit for overall healthcare costs (266, 340–342).

### Strategies to Combat Antimicrobial Resistance

Microbes have been successfully existing on this planet for 4 billion years. The microbial community has faced and prevailed over all types of catastrophes all over the globe, and they have magnificently persisted. These microscopic creatures have efficiently proved over time that they are the survival of the fittest (343). AMR is a natural complex phenomenon, but there are several different causative influences which include the irrational use of antimicrobials (10–13, 344). The fight against AMR needs to combine a global, nationwide, and local line of attack strategy, including the promotion of rational use of antibiotics discussed above.

Less infection leads to less demand for antibiotics. Simple measures, like the cultivation and nurturing of hand hygiene routines, play a part in the effective strategies to prevent and control infections. The battle against AMR needs collective effort, multidisciplinary approaches, and ongoing supervision and monitoring policies (344). The Global Alliance for Infections in Surgery advised seven stratagems to avert hospital and community-acquired infections (345). Those policies are “patient safety; following guidelines; antibiotic stewardship; surveillance; screening patients; environmental hygiene, and hand hygiene (345).”

Some of the WHO recommended approaches include increased collaboration between governments, non-governmental organizations, professional groups, and international agencies; new networks to undertake the surveillance of antimicrobial use and AMR; a global approach to the control of counterfeit antimicrobials; incentives for the research and development of new drugs and vaccines; and forming new or reinforcing existing programs to contain AMR (344).

We have earlier in this review drawn attention to the difficulties that research scientists and pharmaceutical companies face in introducing new antimicrobial drugs to overcome resistance problems. Each endeavor requires time for researchers to overcome the scientific challenges of microbes' rapidly evolving resistance mechanisms, particularly those which involve reduced cell-wall permeability and efflux pumps with broader substrate specificity linked to their cytoplasmic membranes (162, 346, 347). It has been estimated that it takes more than 6–8 years of research effort to identify a new antibacterial active against Gram negative pathogens harboring permeability and efflux pumps (348, 349). Add to this 3–4 years for completing regulatory toxicology studies, non-clinical PK/PD studies for arriving at human dose, and elucidating new drug's DMPK features, and a clinical development phase of another 4–5 years, and the residual patent life may be too short to recoup development costs, Thus, difficult-to-overcome AMR mechanisms and lack of financial incentives combine against new drug introduction and hence the WHO call for incentives for novel research.

Strategies to extend the beneficial therapeutic lifespan of antimicrobials currently available include maintaining the heterogeneity of antimicrobial agents, assuring adequate serum drug concentrations, repurposing of withdrawn and underused antimicrobial drugs, and combination therapy (350). Another possible approach to rejuvenate existing antimicrobials is to explore further combination drug therapy.

Other contemporary novel approaches include (a) bacteriophage therapy, fecal microbiota transplantation, and antimicrobial adjuvants to combat antimicrobial resistance (350, 351).

## Conclusions

Antimicrobial resistance is a multifaceted problem that affects almost all communities and is driven by many interrelated factors. Single isolated attempts or interventions have a limited effect. Therefore, coordinated strategies and actions are required from various stakeholders at the global level in order to curtail the emergence and spread of antimicrobial resistance. Our key findings are summarized in Table 5.

**Table 5:** Key findings of this review.

• The “golden age” of antibiotic discovery, produced many highly useful antimicrobials, some of which are still in use. Over time microbes have developed to all these drugs • Antimicrobial resistance ranks amongst the world most important health problems causing misery, morbidity, and death around the globe • The financial burden of AMR is high as both direct and indirect health costs are increased • Innovative approaches to antimicrobial therapy will continue. However, for the foreseeable future we should not rely on any new ‘magic bullet' to overcome AMR • Shortcomings in undergraduate and postgraduate education and training significantly exacerbate the AMR problem by way of ineffective and imprudent prescribing • Strategies to improve antibiotic prescribing and consumption require educational, managerial, regulatory, and economic approaches in combination • Collaboration between governments, agencies, academia, pharmaceutical industry, healthcare professions, and the community is crucial in combating AMR.

## Recommendations

- Recognition of AMR as an issue of first rank importance to each and every nation is the starting point
- The causes of AMR are multi-factorial and the approaches to combat it must be multipronged and collaborative.
- Stakeholders must work together to combat AMR, targeting different measures at international, national, community, hospital, individual, and patient levels.
- Regulatory agencies have a key role to play in abolishing the availability of antibiotics without prescription and overseeing prescribing of antibiotics that is concordant with national and local formularies.
- Prescribing doctors must be better prepared for their role by “real-world” undergraduate training in prescribing and by locally appropriate continuing professional development.
- Publicity campaigns directed at consumers must be directed at reducing their pressure for inappropriate antibiotic prescribing
- International collaboration is required to support development of the next generation of innovative antimicrobial drugs.

## Limitations of This Study

This paper has all the inherent limitations of narrative reviews. We have tried to counter subjective bias by our wide diversity of authorship from developed and developing nations. The review has benefitted from detailed and thoughtful pre-publication reviews that have strengthened the perspectives we offer.

Systematic reviews and meta-analyses offer valuable syntheses of evidence to policy makers. However, narrative reviews like this one can make an important contribution on a topic as broad as antimicrobial resistance. We have been able to combine a historical perspective on drug development with a forward-looking evaluation of prospects. We have selected evidence from across the world, including both developed and developing countries, as we tried to offer readers a chance to consider where the problems and solutions are universal, and where local factors predicate local solutions.

The literature searches were conducted in a location which offered somewhat limited access to reference sources. However, this has the benefit of making it more likely that readers from around the world will be able to access the primary references, if they so wish.

## Author Contributions

TI and MH: conceptualization. SD, NAAR, EP, MR, MS, MAH, TI, SI, and MH: writing—original draft preparation and supervision. SD, NAAR, EP, MR, MS, MAH, TI, SI, and MH: writing—review and editing and project administration. All authors contributed to the article and approved the submitted version.

## Conflict of Interest

The authors declare that the research was conducted in the absence of any commercial or financial relationships that could be construed as a potential conflict of interest.
